# Self-Biased Magneto-Electric Antenna for Very-Low-Frequency Communications: Exploiting Magnetization Grading and Asymmetric Structure-Induced Resonance

**DOI:** 10.3390/s24020694

**Published:** 2024-01-22

**Authors:** Chung Ming Leung, Haoran Zheng, Jing Yang, Tao Wang, Feifei Wang

**Affiliations:** 1 School of Mechanical Engineering and Automation, Harbin Institute of Technology, Shenzhen 518055, China; 22s153232@stu.hit.edu.cn; 2 Education Center of Experiments and Innovations, Harbin Institute of Technology, Shenzhen 518055, China; yangjing2016@hit.edu.cn; 3 Key Laboratory of Optoelectronic Material and Device, Department of Physics, Shanghai Normal University, Shanghai 200234, China; twang@shnu.edu.cn

**Keywords:** magneto-electric (ME) antennas, self-biased, very low frequency (VLF), digital modulation and demodulation, binary amplitude-shift keying (2ASK), binary phase-shift keying (2PSK)

## Abstract

VLF magneto-electric (ME) antennas have gained attention for their compact size and high radiation efficiency in lossy conductive environments. However, the need for a large DC magnetic field bias presents challenges for miniaturization, limiting portability. This study introduces a self-biased ME antenna with an asymmetric design using two magneto materials, inducing a magnetization grading effect that reduces the resonant frequency during bending. Operating principles are explored, and performance parameters, including the radiation mechanism, intensity and driving power, are experimentally assessed. Leveraging its excellent direct and converse magneto-electric effect, the antenna proves adept at serving as both a transmitter and a receiver. The results indicate that, at 2.09 mW and a frequency of 24.47 kHz, the antenna has the potential to achieve a 2.44 pT magnetic flux density at a 3 m distance. A custom modulation–demodulation circuit is employed, applying 2ASK and 2PSK to validate communication capability at baseband signals of 10 Hz and 100 Hz. This approach offers a practical strategy for the lightweight and compact design of VLF communication systems.

## 1. Introduction

In recent years, the very-low-frequency (VLF, 3–30 kHz) communication field has witnessed significant development, primarily driven by the growing demand for efficient and reliable communication systems [1,2,3,4]. This progress is particularly crucial in the field of geological exploration, where challenging environments such as long distances, underwater environments, underground environments and complex geological terrains make VLF communication increasingly important [5,6,7,8,9]. Traditional antennas, constrained by size limitations, struggle to meet the specific requirements of VLF communication, posing a series of challenges to system design and deployment [10,11,12]. As the scope of VLF communication applications expands, existing antenna designs face numerous limitations, restricting transmission distances, increasing physical footprints, escalating costs and leading to considerable power consumption [13,14]. Therefore, the quest for innovative antenna designs and communication system solutions becomes paramount.

To overcome the challenges in the VLF communication domain, magneto-electric (ME) antennas have emerged with unique designs relying on mechanical acoustics-driven mechanisms. These ME antennas exhibit advantages such as a compact design, lightweight construction and low power consumption [15]. In practical applications, these novel ME antennas outperform traditional small-sized antennas, garnering widespread attention. The innovative design principles of ME antennas position them as promising solutions to the constraints faced by traditional antennas in VLF communication. Their ability to provide compact, lightweight and energy-efficient alternatives addresses the evolving demands of communication systems in challenging environments, making ME antennas a focal point of interest and research [3,16,17].

Due to the numerous advantages offered by ME antennas operating in VLF ranges, significant research efforts have recently been devoted to this area. In 2015, Yao et al. [18] proposed a multi-iron antenna based on bulk acoustic wave intermediation. They not only analyzed and derived the lower limit of its radiation quality factor (Q factor) but also developed a one-dimensional multi-scale finite-difference time-domain code to simulate the proposed antenna structure. The feasibility of their method was verified through numerical calculations and simulation analyses. In 2017, Nan et al. [18] reported on acoustically actuated nanomechanical ME antennas featuring a suspended ferromagnetic/piezoelectric thin-film heterostructure. This marked the introduction of the experimental prototype of ME antennas for the first time. In 2019, Xu et al. [19] introduced a VLF transmitter with a magneto-elastic coupling heterostructure and optimized its structural design. Their work revealed a near field resembling a magnetic dipole through prototype measurements, thereby validating its transmission capabilities. In 2020, Dong et al. [20] proposed a VLF communication system utilizing a pair of ME antennas. They not only studied the radiation mechanism of ME antennas but also verified it through near-field radiation patterns. The distribution of the radiation field with distance was predicted with an analytical model and confirmed via experimentation, successfully demonstrating direct antenna modulation (DAM) on ME antennas. Advancing the field in 2023, Wu et al. [21] proposed a VLF ME antenna driven by the synergistic effect of piezo-driven magnet motion and inverse magneto-electric effects. This innovative approach utilized an electromagnetic coil as the receiving end, showcasing a 10 Hz VLF communication test based on amplitude-shift keying (ASK) and frequency-shift keying (FSK) digital signal modulation. In the aforementioned studies, it can be consistently observed that achieving an optimal radiation intensity for ME antennas often requires a direct current (DC) biased magnetic field [10]. Regardless of whether coils or permanent magnets are used to provide this magnetic field, it results in an increase in the volume and mass of ME antennas, posing challenges for miniaturization and lightweight design [10,22,23]. These challenges underscore the critical need for ongoing innovation in the pursuit of efficient and compact ME antenna designs in VLF applications. In comparison to research development in magneto-electric composites, the development of magnetic field-biased magneto-electric (ME) antennas is relatively new. Recently, a method leveraging the exchange bias effect was employed [24]. It involves the interaction between annealed Metglas, which exhibits “hard” ferromagnetic characteristics, and non-annealed Metglas, which exhibits “soft” ferromagnetic characteristics. As a result, a substantial ME response is achieved without the need for a magnetic field bias. Specifically, since magnetostriction originates from magnetization variation (magnetic domain rotation and domain wall migration), the exchange bias effect results in a shift in the position of magnetostriction, leading to non-zero magnetostriction with zero magnetic field bias [25].

In this work, we propose a self-biased ME antenna pair based on the magnetization grading effect, which can maintain a strong magneto-electric response even in an unbiased state. Compared to the aforementioned self-biased ME antenna, our proposed self-biased ME antenna does not utilize ‘hard’ ferromagnetic materials. Instead, two ferromagnetic materials with different saturation magnetization values are employed to generate a magnetization grading effect, thereby obtaining a magneto-electric response. Essentially, a different effect is utilized to achieve a self-biased outcome. Moreover, this approach eliminates the need for annealing or any specific treatment of the materials themselves to achieve the self-biasing effect. In addition, with both direct and converse magneto-electric effects, the antenna can function as both a transmitter and a receiver [26,27]. The designed ME antenna operates at a resonance frequency (*f*) of 24.47 kHz, exhibiting characteristics of a small volume, light weight and low driving power. The principle of the self-biasing effect was analyzed, and the radiation performance, driving power and scattering parameters of the ME antenna were characterized. Modulation and demodulation circuits were designed, and binary amplitude-shift keying (2ASK) and phase-shift keying (2PSK) communication experiments were conducted with baseband signals at 10 Hz and 100 Hz, demonstrating the feasibility of wireless communication using the proposed VLF ME antenna.

## 2. Fabrication and Characterization

**Figure 1:**
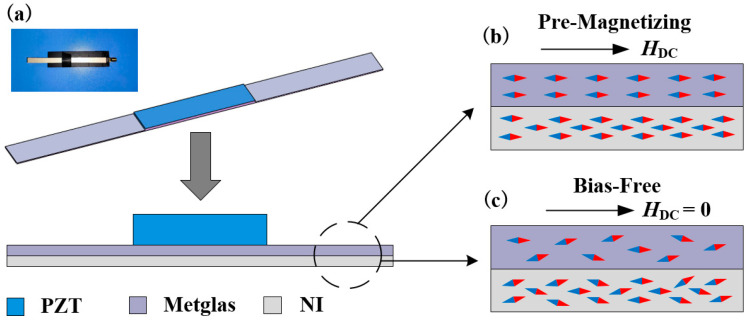
(**a**) Structural illustration and experimental prototype of the finished self-biased ME antenna. (**b**) Distribution diagram of the magnetic moment within the antenna during pre-magnetization (*H*_DC_). (**c**) Distribution diagram of the magnetic moment within the antenna after debiasing (*H*_DC_ = 0).

Figure 1a illustrates a schematic diagram of our proposed self-biased ME antenna, comprising a piezoelectric ceramic (PZT), high-magnetic-permeability magnetostrictive foils (Metglas) and nickel foils, which were all commercially sourced materials. The PZT ceramic plate measured 30 mm × 6 mm × 0.5 mm, with electrodes applied to its upper and lower surfaces. Metglas foils, which were 25 μm thick, and nickel foils, which were 10 μm thick, were each cut into 100 mm × 6 mm dimensions. These materials were stacked sequentially, with five layers of Metglas foils and ten layers of nickel foils, bonded by epoxy resin (West system 105/206) to one side of the PZT plate, forming an asymmetric structure. The self-biasing effect refers to a phenomenon observed in certain materials where, due to their internal magnetic structure or unique magnetic properties, the material generates a self-induced bias magnetic field effect even in the absence of an external magnetic field. This effect allows the material to maintain a certain degree of magnetization without external assistance [28,29]. In detail, the magnetization grading effect arises when two or more dissimilar magnetic materials are bonded together. The occurrence of magnetization grading is due to the combination of material components with different saturation magnetization strengths, resulting in an internal magnetic field. When two different magnetic materials with distinct saturation magnetization strengths are bonded together, an internal magnetic field (*H*_int_) is generated that is anti-parallel to the magnetization intensity gradient (∇*M*). This results in an enhanced magnetostrictive effect [30].

In the proposed ME antenna in this paper, the self-biasing effect is achieved through the combination of Metglas and nickel magnetic materials to create a magnetization grading effect. In the absence of any pre-magnetization, the self-biasing effect is generally weak due to the relatively low strength of the internal magnetic field (*H*_int_). Therefore, the pre-magnetization process is essential, and our pre-magnetization field is applied by placing neodymium iron boron permanent magnets at a certain distance. As shown in Figure 1b,c, under the influence of the pre-biased DC magnetic field (*H*_DC_), both materials exhibit magnetization aligned in the same direction as the applied DC magnetic field, despite having different saturation magnetization values. Upon removing the DC bias magnetic field (*H*_DC_ = 0), the higher saturation magnetization of the NI foil results in a higher residual magnetization, increasing the magnetization gradient. This leads to the generation of an enhanced internal magnetic field (*H*_int_).

In summary, the varying saturation magnetization strengths of nickel and Metglas create a distinction in residual magnetization during pre-magnetization, inducing magnetization grading effects within the composite structure [30]. The deliberate engineering of a self-biasing effect through magnetization grading represents a novel and innovative approach, providing a deeper understanding of the underlying mechanisms governing the antenna’s functionality. The described asymmetric structure and its self-biasing characteristics lay the foundation for further advancements in ME antenna technology, offering enhanced magnetostrictive responses for various applications.

**Figure 2:**
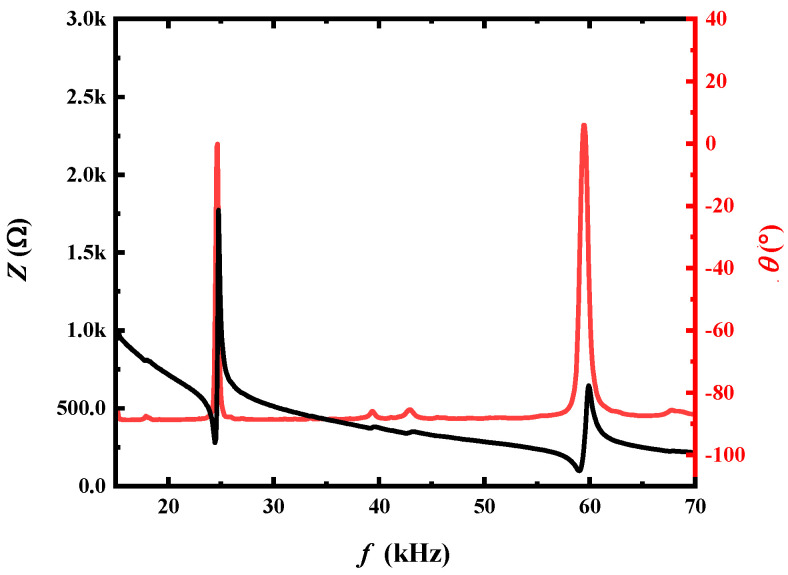
Impedance (*Z*) and phase (*θ*) spectra of the proposed self-biased ME antenna.

Figure 2 depicts the impedance and phase spectra of the ME antenna as measured with the impedance analyzer (Keysight, Santa Rosa, CA, USA). It can be observed that the ME antenna exhibited two resonances. This was attributed to the designed asymmetrical structure, which resulted in the ME antenna having resonance points corresponding to both a bent vibration mode and a length vibration mode. The resonance frequency associated with the bent vibration mode was lower than the resonance frequency of the length vibration mode [2]. In previous studies, the resonance frequency was found to strongly depend on the size effect of the ME composite [7]. Traditionally, achieving a lower resonance frequency required a larger size or volume of the composite, posing significant constraints to antenna design and portability. Notably, the bent vibration mode proposed in this study offered the advantage of maintaining the same size while achieving a lower resonance working frequency. This innovation mitigated the need for a larger volume, overcoming a major limitation in antenna design for portable applications. Consequently, the operating frequency of our proposed antenna was shifted to the VLF band, with the resonant frequency (f) measured at 24.47 kHz. The significance of this lies in the potential to optimize electromagnetic radiation by configuring the operating frequency of the ME antenna around this electromagnetic resonance (EMR) frequency. This finding emphasizes the practical implications of the bent vibration mode in achieving desired resonance characteristics without compromising the size and portability of the antenna.

**Figure 3:**
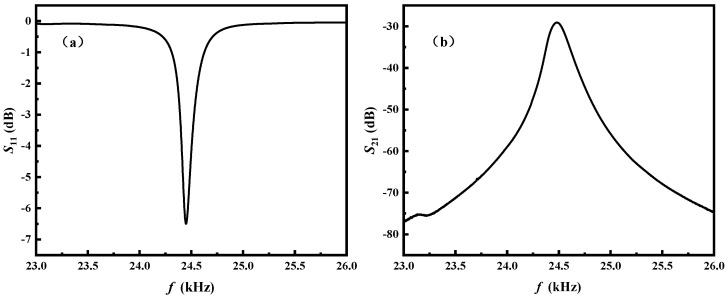
(**a**) Spectra of reflection coefficient (*S*_11_) for the self-biased ME antenna. (**b**) Forward transmission coefficient (*S*_21_) for the self-biased ME antenna.

In the realm of electrical devices, scattering parameters (S-parameters) serve as a crucial tool for characterizing the intricate input–output dynamics between different ports. In this context, the *S*_11_ parameter takes center stage, as it elucidates the input reflection coefficient, more commonly referred to as the input return loss. By quantifying the extent of the signal reflection at the input, the *S*_11_ parameter provides a fundamental insight into the behavior of the antenna. Conversely, the *S*_21_ parameter assumes significance as the forward transmission coefficient, shedding light on the transmission loss experienced by the antenna during signal propagation [31,32]. Understanding these parameters becomes particularly vital when dealing with devices like the ME antenna, designed to operate within specific frequency bands. In the case of our ME antenna developed for operation in the VLF band, a Vector Network Analyzer (Rohde & Schwarz, Munich, Germany) emerged as a pivotal instrument for characterizing its performance. Figure 3a provides a compelling visual representation, showcasing a noteworthy downward peak of 6.5 dB for the *S*_11_ parameter. This significant observation at a frequency of 24.47 kHz implies that approximately 77.6% of the incident power was successfully transmitted by the antenna, underscoring its efficiency in managing signal reflections. Further emphasizing the antenna’s prowess, Figure 3b reveals a substantial upward peak of 29.1 dB for the *S*_21_ parameter, also at a frequency of 24.47 kHz. This peak signifies a remarkable transmission efficiency achieved by the proposed ME antenna, indicating its capability to efficiently propagate signals within the targeted VLF band. The nuanced analysis of these scattering parameters not only validated the effectiveness of the ME antenna but also contributed valuable insights for optimizing its performance in practical applications within the specified frequency range.

## 3. Radiation Performance Characterization of ME Antennas

To determine the maximum achievable propagation distance of the VLF electromagnetic waves produced by our proposed ME antenna, it was necessary to test the distribution of the magnetic field with distance. Due to the far-field region (*r* << λ/2π, where *λ* is the electromagnetic wave wavelength and *r* is the distance) exceeding 2 km at the resonant operating frequency of the ME antenna, it was challenging to test the far-field radiation characteristics. Therefore, we focused solely on the study of near-field radiation to understand the radiation performance of the proposed ME antenna.

**Figure 4:**
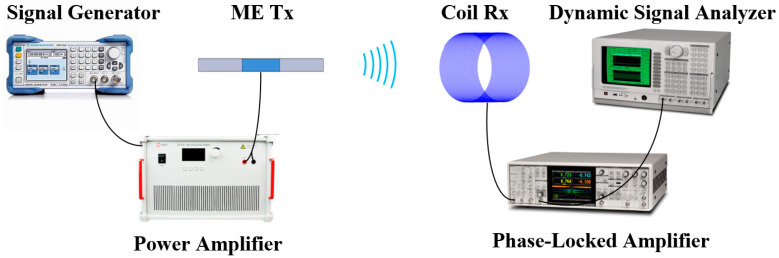
Testing platform for assessing the transmission capabilities of the self-biased ME antenna.

In this setup, a pre-calibrated copper coil functioned as a magnetic field sensor to gauge the strength of the magnetic field signal transmitted by our proposed ME antenna. The copper coil was wound with 300 turns, possessing a diameter of 10 mm and a length of 20 mm. Furthermore, the magnetic detection sensitivity of the copper coil underwent calibration using an in-house automated magnetic field coefficient measurement system [33]. It is worth noting that the utilization of the calibrated copper coil as a magnetic field sensor ensured precise and reliable measurements, contributing to the accuracy and reproducibility of the experimental results. As shown in Figure 4, the excitation signal at a frequency of 24.47 kHz was generated from a signal generator. This signal was then amplified by a power amplifier to a voltage of 1 Vrms, and the amplified voltage drove the piezoelectric phase of the ME antenna. Specifically, the piezoelectric phase generated mechanical stress and strain based on the converse piezoelectric effect. The stress and strain were transferred to the magnetostrictive phase, and subsequently, the magnetic field was generated based on the converse magnetostrictive effect [34]. This combined phenomenon is referred to as the converse magneto-electric effect [35,36]. As observed in the impedance measurement of Figure 2, an impedance of 478e^−j1.2^ for the ME transmitter antenna at 22.47 kHz resulted in an input power of 2.09 mW.

**Figure 5:**
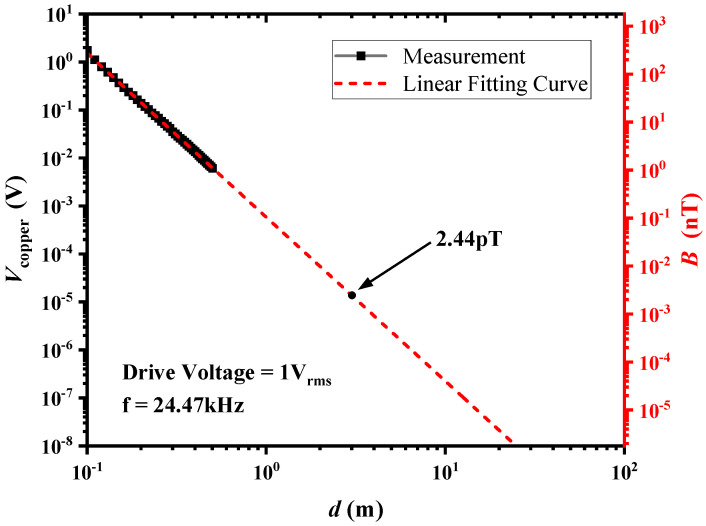
Relationship between electromagnetic radiation intensity and testing distance, with experimental data fitted to a curve for prediction.

During the measurement process, the distance (*d*) between the copper coil and ME transmitter antenna was changed in the range of 0.1–0.5 m, and the transmitted magnetic field was measured using the voltage output (*V*_copper_) captured by our aforementioned pre-calibrated copper coil. At the reception end, the transmitted signal from the ME antenna was captured by the same pre-calibrated copper coil. The output signal at the operating frequency from the copper coil was then amplified using a lock-in amplifier. Finally, the output voltage was acquired and stored by a dynamic signal analyzer, and the detection magnetic field was calculated according to the predetermined magnetic detection sensitivity. To assess the electromagnetic radiation limitations of the antenna, a curve-fitting analysis was performed to determine the electromagnetic radiation intensity at extended distances. We projected the radiation intensity of the ME antenna at 3 m (the distance for the near-field signal transmission experiment). Considering the nonlinear behavior of radiation intensity with increasing drive power, we chose to characterize the radiation intensity at the experimental distance using unit voltage excitation. The test results, presented in Figure 5, reveal a rapid attenuation of the electromagnetic waves generated by the ME antenna in the near field. At a distance of approximately 3 m (by curve fitting) in the air, the magnetic flux density reached about 2.44 pT.

**Figure 6:**
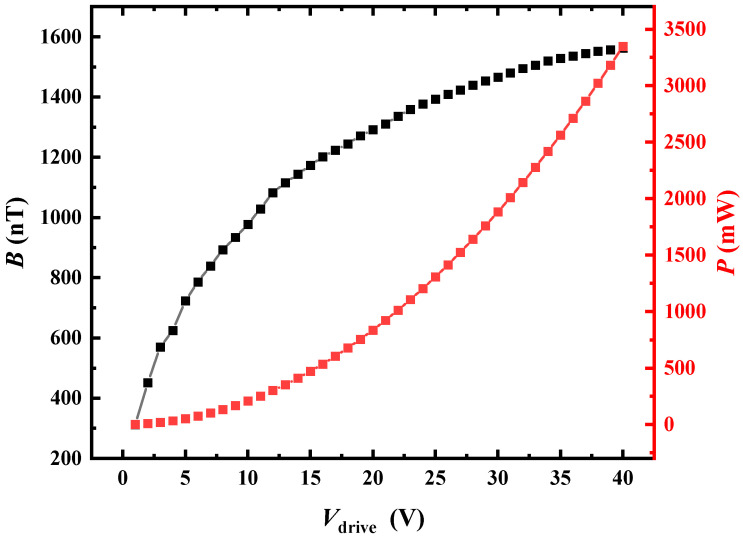
Relationship between electromagnetic radiation intensity and power consumption (driving voltage).

To comprehensively assess the radiation capability, it was crucial to characterize the emitted magnetic field of the ME antenna under varying power levels. The aforementioned copper coil was positioned 0.1 m away from the radiation source of the ME antenna, driven by a voltage ranging from 0 to 40 Vrms at its resonant frequency. In Figure 6, the relationship between radiation field strength and power consumption is depicted as a function of the driving voltage. It is evident that both the radiation field strength and power consumption increased with the driving voltage, but the growth was non-linear. As the driving voltage reached 40 Vrms, the increase in the intensity of the radiation magnetic field noticeably slowed down, displaying a saturation effect. This phenomenon may be attributed to the inability of induced strains in the magnetostrictive phase of magnetic dipole oscillations to continuously increase.

Unlike ME heterostructures operating in quasi-static control, resonant ME devices based on the converse ME effect encounter nonlinear frequency response issues at high vibration amplitudes. Specifically, the fundamental reason is that a higher input power generates larger vibration amplitudes and coupling stress. For widely used ferromagnetic materials such as Metglas and Terfenol-D, their Young’s modulus significantly responds to external stress and magnetic fields; this phenomenon is known as the Delta-E effect. Therefore, from a mechanical perspective, the ME antenna constitutes a nonlinear vibrational system [7]. Therefore, considering the nonlinear behavior of the ME antenna, in the subsequent VLF communication tests, a driving voltage of 40 Vrms was chosen to operate the ME antenna.

## 4. Near-Field Signal Transmission Experiment Based on the Self-Biased ME Antenna

**Figure 7:**
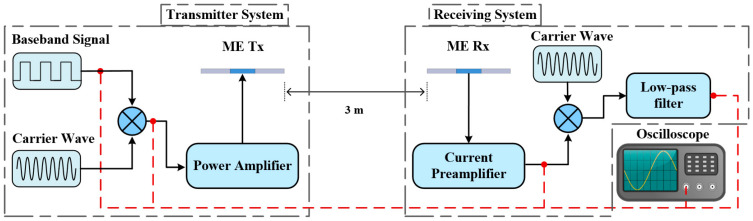
Test platform for a VLF communication system featuring a self-biased magneto-electric antenna pair.

To validate the application of the proposed ME antenna in VLF communication, we established a VLF communication test platform for signal modulation experiments. The test platform was primarily divided into two parts: the ME transmitter integrated setup and the ME receiving integrated setup. The transmitter setup comprised the ME transmitter antenna and the signal modulation system that converted the desired digital signal to a modulated signal. The hardware equipment used in this testing platform was similar to that shown in Figure 4. Digital modulation offers numerous advantages over analog modulation, including better anti-interference performance, stronger resistance to channel loss and enhanced security. We selected two digital modulation schemes for this study: 2ASK and 2PSK. For 2ASK modulation signals, as depicted in Figure 7, modulation of the 2ASK signal was achieved by multiplying the baseband bit with the high-frequency carrier (24.47 kHz sine wave), followed by amplification through a power amplifier before transmission. Finally, the amplified modulated signal was directly loaded onto the ME transmitter antenna. Although the 2PSK and 2ASK modulation methods share fundamental principles, they exhibit key differences. Specifically, 2ASK typically employs bipolar return-to-zero codes, whereas 2PSK uses bipolar non-return-to-zero codes as baseband symbols.

**Figure 8:**
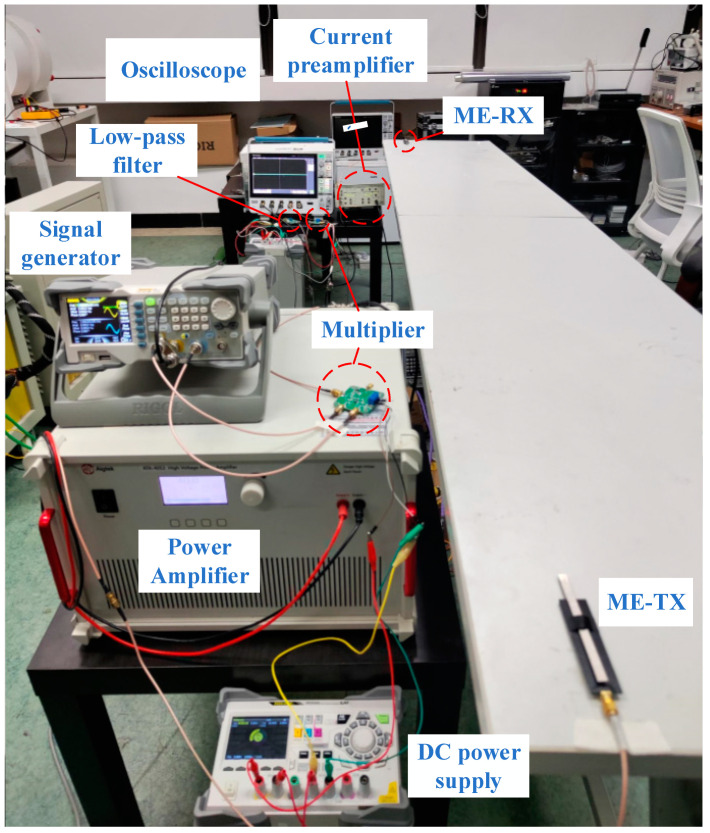
Test platform for a VLF communication system featuring a self-biased ME antenna pair.

The receiving device comprises the ME receiving antenna and the signal demodulation circuit. Upon receiving the electromagnetic signal from the ME transmitter antenna, the ME receiving antenna amplifies it through a low-noise charge preamplifier and processes it through the demodulation circuit back to the desired signal. In comparison to non-coherent demodulation, coherent demodulation can yield higher demodulation efficiency and lower error rates. Therefore, we chose coherent demodulation to process the received signals. Coherent demodulation shares similarities with modulation principles, as both involve frequency spectrum shifting. It necessitates providing a receiver with a carrier signal perfectly synchronized with the modulated signal. After multiplying the received signal by this synchronized carrier signal, a low-pass filter is employed to extract the low-frequency components. The low-frequency component extracted through the low-pass filter is indeed the original baseband signal. Figure 7 and Figure 8 illustrate a schematic diagram and photo of the VLF communication system we established, showcasing all the devices and circuit modules with real-time signal monitoring connected to the oscilloscope displaying the output. In this experiment, the communication test distance was set to 3 m, and the driving voltage was 40 Vrms. This configuration allowed us to assess the performance of the coherent demodulation process under laboratory application conditions, providing valuable information about its efficiency and error rates in a close-to-real application environment.

**Figure 9:**
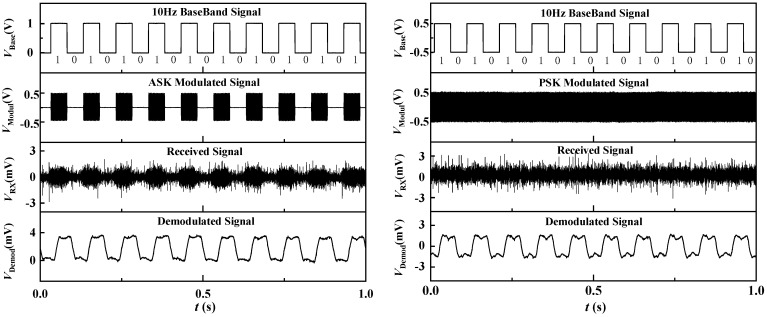
Waveforms of signals measured at each transmission stage at a frequency of 10 Hz under 2ASK and 2PSK modulation schemes.

**Figure 10:**
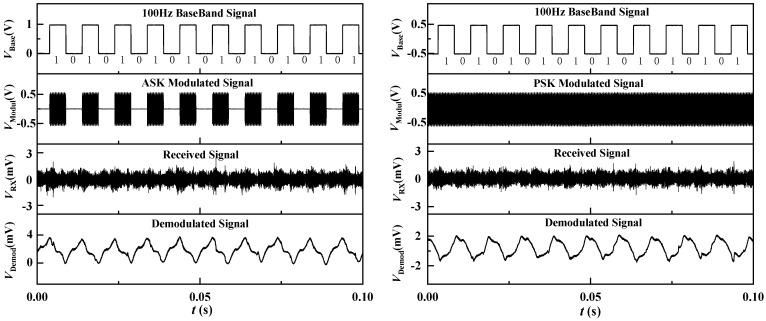
Waveforms of signals measured at each transmission stage at a frequency of 100 Hz under 2ASK and 2PSK modulation schemes.

The experimental setup demonstrates the practical application of our coherent demodulation approach in a VLF communication system. In Figure 9 and Figure 10, arranged from top to bottom, we showcase the baseband signals (*V*_Base_), modulation signals (*V*_Modul_), received signals (*V*_RX_) and demodulated signals (*V*_Demod_) for both 2ASK and 2PSK modulation schemes. When a 10 Hz baseband signal was transmitted, binary modulated signals were successfully received and demodulated into the original bitstream without distortion. Both the 2ASK and 2PSK modulation methods exhibited low waveform distortion, indicating compatibility between the ME antenna and both modulation techniques.

Subsequently, we increased the signal transmission rate by raising the baseband signal frequency to 100 Hz. The experimental results for both modulation schemes are depicted in Figure 10. From the demodulated signals, it is evident that the 2PSK modulation scheme exhibited lower waveform distortion and better waveform quality. This superiority was attributed to the fact that changes in phase information are generally more discernible in a noisy environment, providing PSK with enhanced noise resistance. Conversely, ASK primarily relies on amplitude information, making it more susceptible to channel fading and interference, resulting in degraded signal quality and increased error rates. Consequently, in the context of digital modulation with the ME antenna, 2PSK outperformed 2ASK. Increasing the frequency of the baseband signal enhances the data transmission rate; however, this adjustment results in a shorter signal period, rendering it more susceptible to channel noise and interference. As a consequence, signal distortion, jitter and an increase in error rates may occur. Experimental comparison revealed that, with an elevation in baseband frequency, the demodulated signal waveform underwent noticeable distortion. This experimental outcome underscores the critical importance of selecting an appropriate modulation scheme and frequency in digital modulation schemes, particularly within the VLF communication environment. Optimizing the modulation scheme proves instrumental in enhancing the stability and reliability of data transmission. Furthermore, exploring higher-order modulation methods to improve spectral efficiency emerges as a promising avenue for future research in this domain. The findings underscore the nuanced interplay between the modulation scheme, baseband frequency and overall performance in VLF communication, offering valuable insights for the design and implementation of communication systems in challenging environments.

## 5. Conclusions

In conclusion, a low-frequency communication system was presented, employing a pair of self-biased ME antennas. In contrast to other ME antennas, the elimination of the need for a DC magnetic bias field in our self-biased ME antennas offered a novel approach to miniaturization and lightweight design. The analysis included an examination of the self-biasing phenomenon resulting from magnetization grading effects and the performance of scattering parameter tests on the ME antennas. The distribution of the radiation field intensity with distance under unit drive voltage was scrutinized through experiments, allowing the prediction of the transmission distance based on the self-conducted platform. Furthermore, an analysis of antenna power consumption was conducted. Taking into account the saturation trend of the radiation field intensity with an increasing drive voltage, along with factors such as power consumption and loss, a drive voltage of 40 Vrms was determined for subsequent VLF communication tests. Through this series of analyses, a profound understanding of the working mechanism and performance characteristics of the antenna was gained. This not only facilitates the optimization of the antenna design and the improvement of its performance but also establishes the basis and guidance for further research and applications. Finally, signal transmission tests for a VLF communication system were demonstrated, utilizing our proposed self-biased ME antennas and employing 2ASK and 2PSK digital signal modulation techniques. The results indicate that both modulation schemes exhibited good adaptability. As the baseband signal frequency increased, 2PSK emerged as the more suitable modulation scheme for ME antennas due to its superior noise resistance characteristics. This study provides useful evidence and experience for the design and application of a low-frequency communication system and highlights the potential application prospect of self-biased ME antennas in the VLF communication field.

## Data Availability

Data supporting reported results can be provided upon request.
